# Novel tapered-and-flared fully covered self-expandable metal stent for distal bile duct obstruction

**DOI:** 10.1055/a-2325-2559

**Published:** 2024-06-05

**Authors:** Makoto Kobayashi, Hiroki Kato, Shintaro Tominaga, Motoyoshi Yano

**Affiliations:** 1 Department of Gastroenterology, Yokkaichi Municipal Hospital, Yokkaichi, Japan

**Fig. 1:**
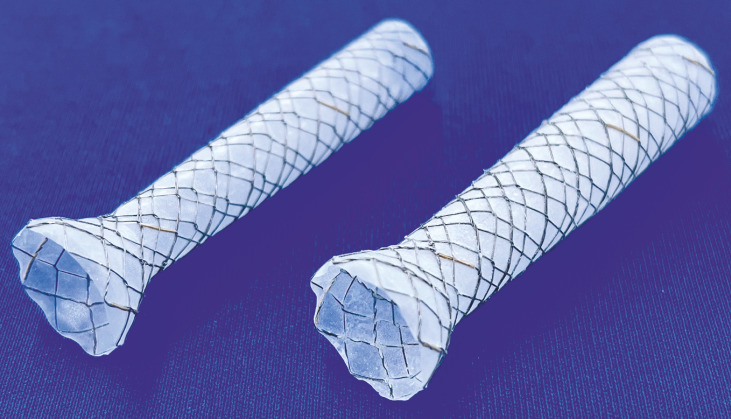
Tapered-and-flared fully covered self-expandable metal stent.

Stent migration is a major problem that can occur when using a fully covered self-expandable metal stent (FCSEMS) in malignant distal bile duct stricture. The K-papilla Stent (S and G Biotech, Yongin, Korea) ( Fig. 1 ) is an FCSEMS with both a tapered portion and flared portion. This stent can be adjusted to the shape of the distal bile duct to the duodenum and has a narrowed papillary portion, which can stabilize its position and prevent migration.

**Fig. 2:**
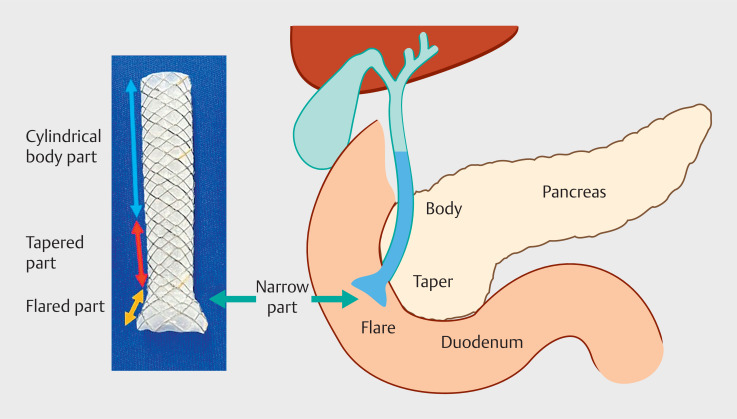
The stent can be adjusted to the shape of the distal bile duct to the duodenum.

**Fig. 3:**
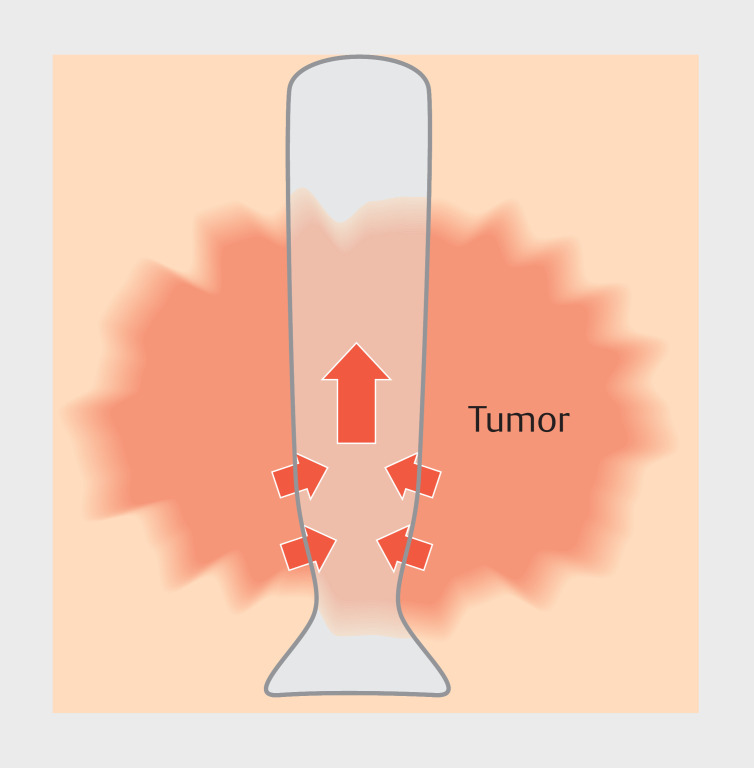
The tapered portion of the stent is subjected to oblique forces by the tumor; thus, the stent will move toward the hilar and be stabilized by the flare.

The stent is fully covered and consists of three parts: a cylindrical body part, a tapered part, and a flared part on the duodenal side ( Fig. 2 ). There is a narrowing of the tapered part as it leads into the papillary portion so that the stent can conform to the shape of the bile duct. The thinnest portion is located at the orifice. The large flare on the duodenal side prevents stent migration into the bile duct. The tapered portion of the stent is subjected to oblique forces by the tumor; thus, the stent will move toward the hilar and be stabilized by the flare ( Fig. 3 ).

A 54-year-old woman was diagnosed with obstructive jaundice due to pancreatic head cancer. After percutaneous bile duct drainage was performed, a tapered-and-flared stent was placed endoscopically ( Video 1 ). The stent was deployed using the yellow marker as a guide and its position was confirmed by X-ray.

The tapered-and-flared stent is unlikely to migrate because its position is fixed based on the shape of the bile duct. Slight misplacement can be corrected spontaneously.Video 1

**Fig. 4:**
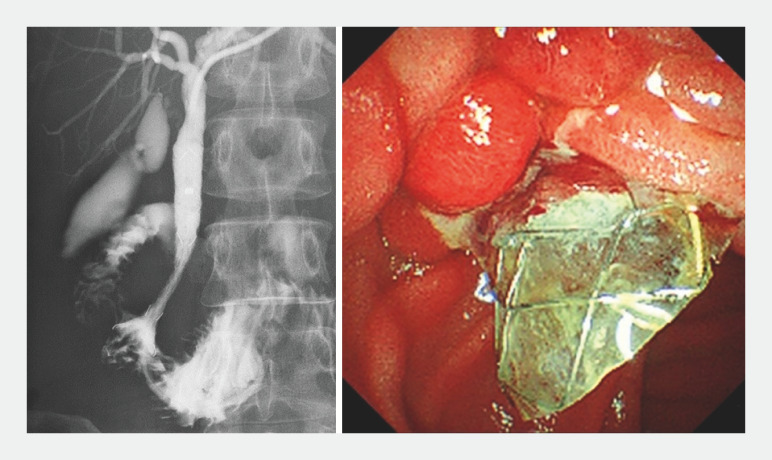
Immediately after deployment, the stent position was slightly longer on the duodenal side.

**Fig. 5:**
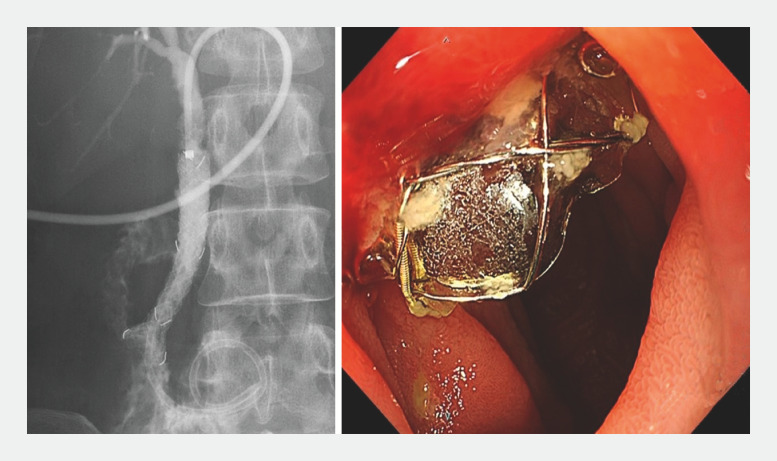
Endoscopy 10 days later showed that the stent had moved toward the liver and was fixed in a stable position.

Immediately after deployment, the stent position was slightly longer on the duodenal side ( Fig. 4 ), but 10 days later, endoscopy showed that the stent had moved toward the liver and was fixed in a stable position ( Fig. 5 ). After stent placement, the patient’s jaundice improved, preoperative chemotherapy was administered, and she underwent a pancreaticoduodenectomy.

Endoscopy_UCTN_Code_TTT_1AR_2AZ

